# The Influence of Media Coverage and Governmental Policies on Google Queries Related to COVID-19 Cutaneous Symptoms: Infodemiology Study

**DOI:** 10.2196/25651

**Published:** 2021-02-25

**Authors:** Solene Huynh Dagher, Guillaume Lamé, Thomas Hubiche, Khaled Ezzedine, Tu Anh Duong

**Affiliations:** 1 Assistance Publique des Hôpitaux de Paris (AP-HP) Département de dermatologie Hôpital Henri Mondor Créteil France; 2 Laboratoire Génie Industriel CentraleSupélec Université Paris-Saclay Gif-sur-Yvette France; 3 Département de dermatologie Centre hospitalier universitaire de Nice Nice France; 4 EA 7379, EpidermE Université Paris-Est Créteil Créteil France; 5 Chaire Avenir Santé numérique Équipe 8 IMRB U955, INSERM Université Paris-Est Créteil Créteil France

**Keywords:** chilblains, COVID-19, dermatology, Google Trends, infodemiology, lesion, media, media coverage, online health information, skin lesions, trend

## Abstract

**Background:**

During COVID-19, studies have reported the appearance of internet searches for disease symptoms before their validation by the World Health Organization. This suggested that monitoring of these searches with tools including Google Trends may help monitor the pandemic itself. In Europe and North America, dermatologists reported an unexpected outbreak of cutaneous acral lesions (eg, chilblain-like lesions) in April 2020. However, external factors such as public communications may also hinder the use of Google Trends as an infodemiology tool.

**Objective:**

The study aimed to assess the impact of media announcements and lockdown enforcement on internet searches related to cutaneous acral lesions during the COVID-19 outbreak in 2020.

**Methods:**

Two searches on Google Trends, including daily relative search volumes for (1) “toe” or “chilblains” and (2) “coronavirus,” were performed from January 1 to May 16, 2020, with the United States, the United Kingdom, France, Italy, Spain, and Germany as the countries of choice. The ratio of interest over time in “chilblains” and “coronavirus” was plotted. To assess the impact of lockdown enforcement and media coverage on these internet searches, we performed an interrupted time-series analysis for each country.

**Results:**

The ratio of interest over time in “chilblains” to “coronavirus” showed a constant upward trend. In France, Italy, and the United Kingdom, lockdown enforcement was associated with a significant slope change for “chilblain” searches with a variation coefficient of 1.06 (SE 0.42) (*P*=0.01), 1.04 (SE 0.28) (*P*<.01), and 1.21 (SE 0.44) (*P*=0.01), respectively. After media announcements, these ratios significantly increased in France, Spain, Italy, and the United States with variation coefficients of 18.95 (SE 5.77) (*P*=.001), 31.31 (SE 6.31) (*P*<.001), 14.57 (SE 6.33) (*P*=.02), and 11.24 (SE 4.93) (*P*=.02), respectively, followed by a significant downward trend in France (–1.82 [SE 0.45]), Spain (–1.10 [SE 0.38]), and Italy (–0.93 [SE 0.33]) (*P*<.001, *P*=0.004, and *P*<.001, respectively). The adjusted R^2^ values were 0.311, 0.351, 0.325, and 0.305 for France, Spain, Italy, and the United States, respectively, suggesting an average correlation between time and the search volume; however, this correlation was weak for Germany and the United Kingdom.

**Conclusions:**

To date, the association between chilblain-like lesions and COVID-19 remains controversial; however, our results indicate that Google queries of “chilblain” were highly influenced by media coverage and government policies, indicating that caution should be exercised when using Google Trends as a monitoring tool for emerging diseases.

## Introduction

The use of social media and search engines for information on diseases and their diagnosis increased considerably in the last decade [1-3]. Infodemiology can be defined as “the science of distribution and determinants of information in an electronic medium, specifically the Internet, or in a population, with the ultimate aim to inform public health and public policy” [4-6]. Queries on search engines including Google and tracking of specific health research terms could constitute an early warning signal for the emergence of diseases, as already shown during the COVID-19 pandemic [7,8].

The first-described COVID-19 symptoms were fever, fatigue, dyspnea, dry cough, and anorexia [9]. Later, physicians described other symptoms associated with the disease, such as anosmia or acral cutaneous lesions [10]. A group of French dermatologists reported an outbreak of these lesions, describing them as erythemas with vesicles or pustules located on extremities (eg, chilblain-like lesions) in paucisymptomatic patients, in a WhatsApp group [11]; subsequently, several studies reported this uncommon cutaneous manifestation without confirming its association with COVID-19 [12,13]. Chilblain-like lesions were the most frequently reported cutaneous findings during COVID-19.

Studies revealed a surge in individual Google searches related to chilblains in early 2020 during COVID-19 [14-16]. Kluger et al [14] and Hughes et al [15] reported an increase in searches during early March in France, the United States, and worldwide, suggesting a potential link between searches on chilblain-like lesions and an increased frequency of such lesions in the context of COVID-19. However, these studies do not consider alternative explanations for surges in these Google searches, concurrent with the curiosity generated by public announcements on new symptoms. Previous studies have highlighted the impact of these events on Google searches during COVID-19 [17]. As opposed to anosmia and other symptoms [18], chilblain-like lesions appear late during the infection, implying that internet queries are more likely to be affected by early media announcements.

To assess the possibility of monitoring the dynamics of COVID-19 by analyzing internet searches, we first need to understand whether the increase in Google searches for chilblains reflect actual symptoms or mere curiosity after public announcements. In particular, in the context of an emerging infectious disease, it is important to investigate the impact of external factors, such as media coverage and strong government policies, on Google queries. Using Google Trends (GT), we sought to study the influence of public announcements related to chilblain-like lesions and government decisions (eg, lockdown enforcement) on searches related to chilblains during COVID-19.

## Methods

### Study Design

We used GT to measure internet searches on chilblain-like symptoms during COVID-19 and used these data to assess the impact of media announcements and government policy decisions on internet search behaviors in accordance with existing guidelines [19].

### Search Strategy

To select keywords, we selected commonly used search terms related to chilblain-like lesions, such as “toe” or “frostbite,” in order to retrieve queries made by the general population. We did not include “finger,” since excessive use of sanitizers during the pandemic resulted in irritation and contact dermatitis. Furthermore, we measured searches of medical terms such as “chilblains” using both the singular and plural forms of the search term. We did not take misspellings into account. Since “toe” can be used in different contexts, we restricted our searches to the “health” category. We used quotation marks for queries including strings of >2 words (eg, “dedo del pie” in Spanish). Search strings included a combination of these terms with the “+” operator denoting the ”OR” logical function (Table 1). “Coronavirus” and “COVID-19” were searched in a separate query, also restricted to the “health” category.

**Table 1:** Search inputs, date of lockdown enforcement, and date of the initial press release on acral lesions in France, Spain, Italy, Germany, the United Kingdom, and the United States.

Data points	France	Spain	Italy	Germany	United Kingdom	United States
Search input	Orteil + orteils + engelure + engelures	sabañones + “dedo del pie” + “dedos de les pies” + erupciones + pies + moretones	gelone + piedi + “dita dei piedi” + geloni	zehen + Hautausschlag + flecken + frostbeulen + erfrierungen	toe + toes + frostbite + chilblain + chilblains + “chill burn”	toe + toes + frostbite + chilblain + chilblains + “chill burn”
Lockdown enforcement date	March 17	March 13	March 9	March 22	March 23	March 21^a^
Press release date	April 6	April 9	April 9	April 20	April 14	April 14

^a^In the United States, we chose March 21 as the date of lockdown enforcement, since this date differed among states: California, March 19; Illinois, New Jersey, and New York, March 22; Ohio, March 23.

We included high-income countries whose populations are familiar with search engines and where Google is the leading search engine. Furthermore, we included countries with different government responses to the pandemic and different announcements related to chilblain-like lesions, including the United States, the United Kingdom, Spain, Italy, France, and Germany. We translated the search terms in the five languages (English, French, Spanish, Italian, and German) using both internet-based translators and support from native speakers.

### Identification of External Factors

First, we hypothesized a change in people's internet behavior immediately after the lockdown enforcement, since the measure was a major disruption to their daily routine. Second, we hypothesized that the first announcement of a potential association between chilblain-like lesions and COVID-19 could have led to curiosity-driven internet searches. Therefore, GT data on acral lesions were assessed and compared through 3 periods: (1) from early 2020 until the lockdown was enforced, (2) from lockdown enforcement to the date when the association between acral lesions and COVID-19 was first reported, and (3) from the date of the first report of the aforementioned association until May 16, 2020.

For each country, we documented the dates of lockdown enforcement and the dates when an association between the acral lesions and COVID-19 was first reported (Table 1). The press releases or scientific communications on chilblain-like lesions are indicated in Table 2.

**Table 2:** First nationwide media release and scientific communication related to chilblain-like lesions in France, Spain, Italy, Germany, the United Kingdom, and the United States.

Country	First chilblain-like lesion–related communication	Date
France	Scientific communication: [20]; press release: [21]	April 6, 2020
Spain	Scientific communication: [22]	April 9, 2020
Italy	Press release: [23,24]	April 9, 2020
United Kingdom	Press release: [25-27]	April 14, 2020
United States	Press release: [28]	April 14, 2020
Germany	Press release: [29]	April 20, 2020

### Generation of the Data Set

GT data from January 1 to May 16, 2020, were extracted for all six countries. We selected January 1 as the starting date to assess baseline interest before the pandemic in Europe, and May 16 because it allowed for data collection before, during, and after the lockdown (varying depending on the government measures in each country).

Day-to-day relative search volume (RSV) data were retrieved from GT on May 19, 2020. RSVs were calculated by GT as follows: search results are proportionate to the time and location of a query. Each data point is divided by the total searches from that geographical region and the time range it represents to compare relative popularity. The resulting numbers of a topic are then scaled 0-100 on the basis of its proportion to all searches on all topics.

### Time-Series Analysis

For each country, the RSVs for acral lesions, COVID-19, and the ratio of the RSVs for acral lesions to those of COVID-19 were plotted from March 1 to May 16, 2020.

To assess the impact of external factors including lockdown enforcement and press releases, we used a segmented linear regression model with the approach described by Lagarde [30]. The dependent variable was RSVs, and the independent variable was time. The dates of lockdown enforcement and press release in each country were included in the model as interventions to evaluate their impact on internet search behavior. Table 3 describes the variables in this model.

The results are expressed as estimated variation coefficient (SE) values. Autocorrelation was expected owing to the nature of the data and confirmed through a Durbin-Watson test. Thus, regression analysis was performed using the Prais-Winsten method, a generalized least squares estimator. Statistical analysis was performed using R software (version 4.0.0, The R Foundation). All tests were two-tailed, and *P*.05 indicated significance.

Media contamination among countries might have occurred herein. People in one country might be aware of chilblain-like COVID-19 symptoms through foreign media before their own national media reported such information. We conducted a sensitivity analysis to assess this phenomenon, using the date of the first public communication on chilblain-like symptoms (April 6, 2020) as the date of the press release (instead of the actual date of the first press release in each country).

**Table 3:** Variables in the segmented linear regression model.

Variables		Description	Details
Dependent variable: relative search volume		Relative search volumes	Extracted from Google Trends
**Independent variables**			
	Time	Secular trend	Number of days since January 1, 2020
	Media announcement	Change in level due to media announcement	Dichotomous variable indicating whether the first media announcement in each country had already occurred
	Time since media announcement	Change in trend due to media announcement	Number of days since the first media announcement in the country (0 before this date)
	Lockdown	Change in level due to the lockdown	Dichotomous variable indicating whether the lockdown was already enforced in each country
	Time since lockdown enforcement	Change in trend due to the lockdown	Number of days since nationwide lockdown enforcement (0 before this date)

## Results

### RSV for COVID-19 or Acral Lesions

**Figure 1:**
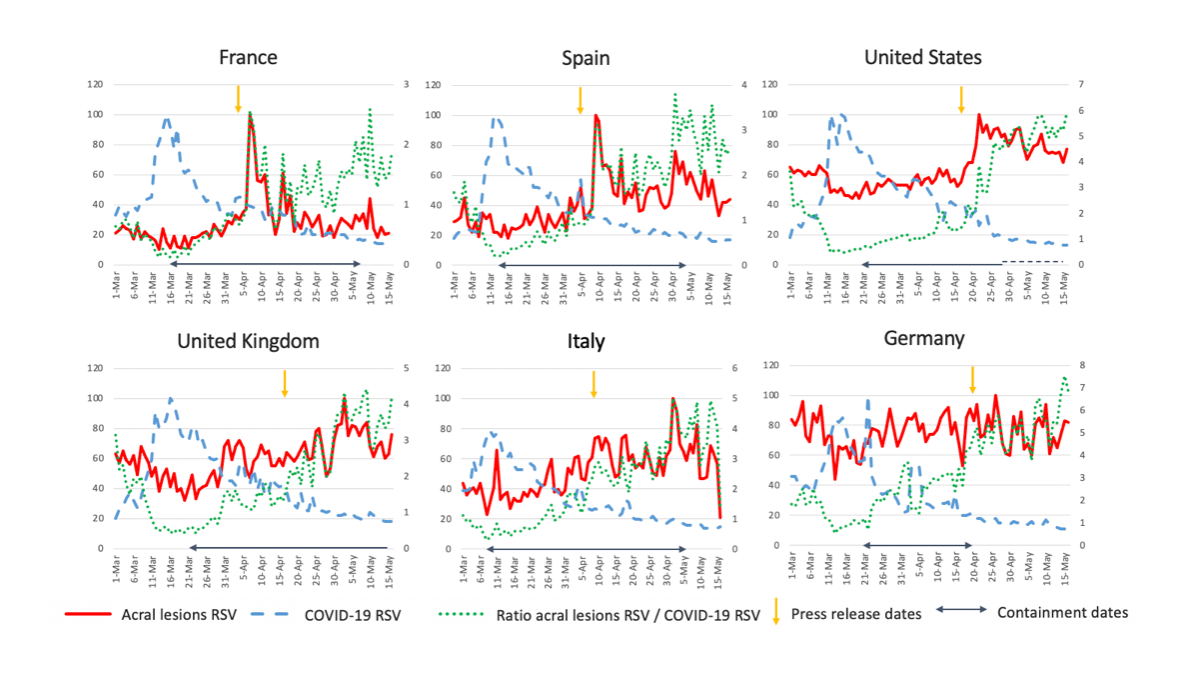
Graphs representing chilblain-like lesion relative search volumes (RSV), COVID-19 RSV, and the ratio of chilblain-like lesion RSV to COVID-19 RSV for France, Italy, Spain, Germany, the United States, and the United Kingdom between March 1 and May 16, 2020. Dates of press release are indicated with yellow arrows.

The frequency of queries for acral lesion–related terms did not vary from baseline values between January 1 and March 1, 2020. COVID-19–related queries increased from March 1 (Figure 1), peaking in all six countries between March 10 and 20, 2020, corresponding to the pandemic outbreak and lockdown enforcement policies in Europe. In three countries (France, Spain, and the United States), acral lesion RSVs peaked immediately after press releases. The ratios between acral lesion RSVs and COVID-19 RSVs show a constant upward trend, suggesting a relative increase in acral lesion–related searches rather than for COVID-19–related searches (Figure 1).

### Interrupted Time-Series Analysis

**Figure 2:**
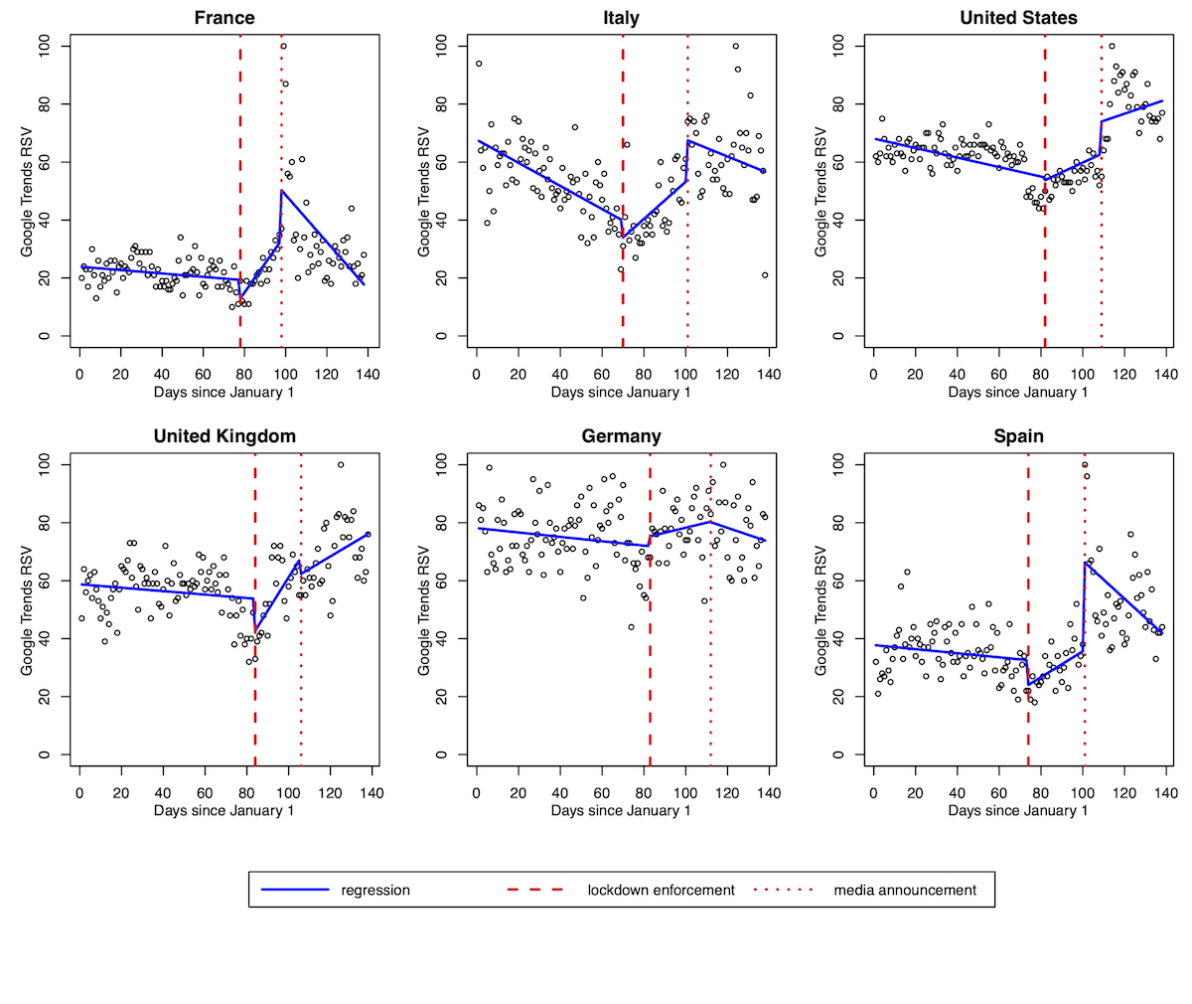
Segmented linear regression model for acral lesion–related relative search volumes. The model integrates the dates of lockdown enforcement and acral lesion–related press releases for France, Italy, Spain, Germany, the United States, and the United Kingdom. RSV: relative search volume.

On segmented linear regression analysis, lockdown enforcement was associated with a significant slope change in acral lesion–related searches in France, Italy, and the United Kingdom, with variation coefficients of 1.06 (SE 0.42), 1.04 (SE 0.28), and 1.21 (SE 0.44), respectively. A nonsignificant upward trend was observed in Germany, the United States, and Spain (Figure 2).

RSVs significantly increased before press releases in France, Spain, Italy, and the United States, with variation coefficients of 18.95 (SE 5.77), 31.31 (SE 6.31), 14.57 (SE 6.33), and 11.24 (SE 4.93), respectively, with no significant changes in Germany and the United Kingdom (0.23 [SE 6.48]; *P*=.97 and –4.99 [SE 6.68]; *P*=.46, respectively). RSVs displayed a significant downward trend after press releases in France, Spain, and Italy (variation coefficients –1.82 [SE 0.45], –1.10 [SE 0.38], and –0.93 [SE 0.33], respectively). In the United States and the United Kingdom, RSVs displayed an upward trend after press releases, albeit in a nonsignificant manner (variation coefficients –0.09 [SE 0.42]; *P*=.83 and –0.72 [SE 0.53]; *P*=.18, respectively).

Adjusted R^2^ values were 0.311, 0.351, 0.325, and 0.305 for France, Spain, Italy, and the United States, respectively, suggesting an average correlation; those for Germany and the United Kingdom were 0.017 and 0.147, respectively, suggesting a weak correlation (Tables 4 and 5).

**Table 4:** Coefficient values and adjusted R^2^ values of the linear segmented regression model integrating dates of nationwide lockdown enforcement and of acral lesion–related press releases in France, Spain, and the United States.

Trends	France			Spain			United States	
	Variation coefficient (SE)	*P* value	Variation coefficient (SE)		*P* value	Variation coefficient (SE)		*P* value
Constant	23.89^a^ (2.65)	<.001	37.77^a^ (3.21)		<.001	68.08^a^ (3.20)		<.001
Secular trend	–0.05 (0.05)	.32	–0.07 (0.07)		.35	–0.16^b^ (0.06)		.01
Change in level after lockdown	–7.45 (5.61)	.19	–9.01 (5.87)		.13	–1.38 (4.63)		.77
Change in trend after lockdown	1.06^b^ (0.42)	.01	0.51 (0.33)		.12	0.49 (0.29)		.09
Change in level after press releases	18.95^b^ (5.77)	.001	31.31^a^ (6.31)		<.001	11.24^b^ (4.93)		.02
Change in trends after press releases	–1.82^a^ (0.45)	<.001	–1.10^b^ (0.38)		.004	–0.09 (0.42)		.83
Adjusted R^2^	0.311	—^c^	0.351		—	0.305		—

^a^*P*<.001

^b^*P*<.05.

^c^Not applicable.

**Table 5:** Coefficient values and adjusted R^2^ values of the linear segmented regression model integrating dates of nationwide lockdown enforcement and of acral lesion–related press releases in the United Kingdom, Italy, and Germany.

Trends	United Kingdom^a^		Italy			Germany	
	Variation coefficient (SE)	*P* value	Variation coefficient (SE)	*P* value	Variation coefficient (SE)		*P* value
Constant	58.76^b^ (3.35)	<.001	67.7^b^ (3.32)	<.001	78.14^b^ (2.77)		<.001
Secular trend	–0.06 (0.07)	.39	–0.39^b^ (0.08)	<.001	–0.07 (0.05)		.20
Change in level after lockdown	–12.24 (6.27)	.05	–6.85 (5.81)	.24	3.21 (5.37)		.55
Change in trend after lockdown	1.21^c^ (0.44)	.01	1.04^b^ (0.28)	<.001	0.24 (0.27)		.37
Change in level after press releases	–4.99 (6.68)	.46	14.57^c^ (6.33)	.02	0.23 (6.48)		.97
Change in trends after press releases	–0.72 (0.53)	.18	–0.93^b^ (0.33)	<.001	–0.41 (0.41)		.31
Adjusted R^2^	0.147	—^d^	0.325	—	0.017		—

^a^A sensitivity analysis was performed considering April 6, 2020, as the date of press release. This modification did not improve the fit for 4 of 6 countries (France, Italy, the United States, and Spain). For Germany, the adjusted R^2^ value increased from 0.017 to 0.018; United Kingdom, from 0.147 to 0.209. We obtained the following estimates for model variables: constant 59.12 (SE 3.05; *P*<.001), secular trend –0.07 (SE 0.063; *P*=.26), change in level after lockdown –20.5 (SE 6.78; *P*=.003), change in trend after lockdown 2.90 (SE 0.75; *P*<.001), change in level after press releases –15.4 (SE 6.63; *P*=.02), and change in trend after press releases –2.34 (SE 0.787; *P*=.003).

^b^*P*<.001

^c^*P*<.05.

^d^Not applicable.

## Discussion

### Principal Findings

Our study shows that GT searches for chilblain-like lesions are influenced by lockdown enforcement and media coverage. Chilblain-like lesion–related searches were the highest in France, Spain, Italy, and the United States, seemingly affected by press releases about an outbreak of acral lesions.

While a downward trend was observed in European countries after the press release, regression analysis revealed an upward trend in the United States. However, visual and regression analyses suggest that the model does not fit the US data well, probably owing to the time parameters selected for the model because the dates of lockdown enforcement, press releases, and disease progression differed among states in the United States. This could also reflect the variations in COVID-19 dynamics nationwide.

In France, the United Kingdom, and Italy, the lockdown policies significantly impacted GT dynamics. Lockdown enforcement did not have a significant impact in other countries. In Germany, neither the lockdown nor press releases influenced individual searches related to acral lesions. In all other countries, individual RSV searches increased before the press release. Simultaneously, the search ratio for acral lesions vs COVID-19 remained high, indicating a high interest in acral lesions rather than in COVID-19.

Studies reporting an increase in public interest in COVID-19 before the outbreak in several countries supported the potential role of GT as a surveillance tool [31]. However, for novel diseases such as COVID-19, media coverage or significant announcements by the World Health Organization may strongly influence generic searches. Studies have reported a correlation between new COVID-19 cases or deaths and queries related to COVID-19 symptoms (specific: fever, cough, or pneumonia; unspecific: anosmia or ageusia). However, media releases regarding new clinical signs also strongly influenced internet searches [32-34]. This calls into question the potential to successfully predict the pandemic by analyzing GT data. Regarding anosmia and ageusia, an increase in RSV searches related to acral lesions preceded public scientific communications [18,35,36]. Our results confirm the generation of public interest before press releases, with a peak corresponding to media coverage in several European countries; however, caution must be exercised when using GT as an epidemiological monitoring tool for new or unknown diseases.

Beyond epidemic monitoring, our results illustrate the use of GT in monitoring the reactions of the general population to health-related communications during a period with a high incidence of anxiety and disrupted health care provision. Spikes in interest generated by public announcements on potential new manifestations of the disease should be matched with appropriate health-related information [37]. Some studies have revealed a change in search patterns during COVID-19 for a broad range of health-related topics, including otorhinolaryngologic, lung, or dermatological diseases [33,38,39]. These queries should be matched with appropriate health-related communication to ensure that people can easily obtain the right information and take appropriate action. The increase in searches for a broad range of health-related topics might also suggest that curiosity and inquisitiveness played an essential role in these internet searches, which would undermine attempts to use GT to identify changes in disease dynamics. Moreover, physicians might have also carried out internet searches for chilblains.

We did not perform correlation analyses with the number of COVID-19–related deaths. Indeed, chilblain-like lesions are associated with mild or asymptomatic SARS-CoV-2 infection [40,41], thus undermining correlation analyses with the number of COVID-19–related deaths, and correlations with the number of detected COVID-19 cases are unreliable owing to the limited number of tests performed at the beginning of the pandemic in many countries. Therefore, caution must be exercised when performing correlation analyses between GT data on chilblain-like symptoms and COVID-19 progression.

### Limitations

This study has some limitations of note. First, the results depend on the quality of the initial GT searches, especially keywords. Query RSVs vary greatly in accordance with the search terms. We attempted to pragmatically use search terms that could be used by medical practitioners (such as “chilblain”) and nonmedical individuals (such as “toes”). It was difficult to identify dermatological keywords for the study. We inquired about the potential terms the general population might have used to search for chilblain-like lesions (showing images of chilblains). Most of our participants with a nonmedical background would have searched “eczema” or “urticaria” on Google. In dermatology, it would be ideal to conduct the same study with individuals clicking on pictures resembling their cutaneous lesions [10].

We used direct language translations and did not include misspellings, accents, and special character variations. Thus, we may have missed certain nuances and additional queries. However, we believe that using only the correct spelling might underestimate the RSV, making our results even more robust. To evaluate the potential limitation due to keywords, we assessed the results of “related queries” (top and rising) for the same dates and regions and compared them with GT search terms. It appears that “coronavirus” or “covid” is often associated with “toe” or “frostbite.” However, we intentionally excluded COVID-19–related keywords in order to capture searches performed by individuals who did not consider a potential association between acral lesions and COVID-19.

Furthermore, our data set is limited to the pandemic's first wave between January and May 2020. Studies using these initial data should be repeated when more data are available to determine whether the results remain applicable for subsequent waves of the pandemic.

In addition, our nationwide analyses did not consider the heterogeneity of the regional effects of the pandemic in these countries. This holds particularly true for Italy and the United States, which have been severely affected by the pandemic (eg, Lombardy and New York state, respectively). Thus, state-level analysis of pandemic hotspots might have yielded more reliable results. In some countries including Germany, the incidence of COVID-19 was relatively low. Another study limitation is our use of only one search engine; nonetheless, Google is the most frequently used internet search engine in these six countries [42].

Moreover, internet users are not necessarily representative of the general population, especially in high-income countries with an increasingly elderly population. However, chilblain-like lesions are observed in young patients, who are more prone to searching information on the internet.

Finally, the pathophysiological link between chilblain-like lesions and COVID-19 remains unclear [16,40,43] and is beyond the scope of this study.

### Conclusions

This study suggests that during the first wave of COVID-19, public announcements and government policies served as driving factors for Google queries related to acral lesions. For emerging diseases, the use of GT to detect new symptoms and monitor disease progression is limited by high media coverage levels and government policies.

## Abbreviations

GT: Google Trends
RSV: relative search volume
